# The role of sense of effort on self-selected cycling power output

**DOI:** 10.3389/fphys.2014.00115

**Published:** 2014-03-31

**Authors:** Ryan J. Christian, David J. Bishop, François Billaut, Olivier Girard

**Affiliations:** ^1^Institute of Sport, Exercise and Active Living, College of Sport and Exercise Science, Victoria UniversityMelbourne, VIC, Australia; ^2^Aspetar - Athlete Health and Performance Research Centre, Qatar Orthopaedic and Sports Medicine HospitalDoha, Qatar; ^3^Départment de Kinésiology, Université LavalQuébec, QC, Canada; ^4^Faculty of Biology and Medicine, Institute of Sport Sciences, University of LausanneLausanne, Switzerland

**Keywords:** perceived peripheral discomfort, ratings of perceived exertion, conscious awareness, complex system regulation, hypoxia

## Abstract

**Purpose:** We explored the effects of the sense of effort and accompanying perceptions of peripheral discomfort on self-selected cycle power output under two different inspired O_2_ fractions.

**Methods:** On separate days, eight trained males cycled for 5 min at a constant subjective effort (sense of effort of ‘3’ on a modified Borg CR10 scale), immediately followed by five 4-s progressive submaximal (sense of effort of “4, 5, 6, 7, and 8”; 40 s between bouts) and two 4-s maximal (sense of effort of “10”; 3 min between bouts) bouts under normoxia (NM: fraction of inspired O_2_ [FiO_2_] 0.21) and hypoxia (HY: [FiO_2_] 0.13). Physiological (Heart Rate, arterial oxygen saturation (S_p_O_2_) and quadriceps Root Mean Square (RMS) electromyographical activity) and perceptual responses (overall peripheral discomfort, difficulty breathing and limb discomfort) were recorded.

**Results:** Power output and normalized quadriceps RMS activity were not different between conditions during any exercise bout (*p* > 0.05) and remained unchanged across time during the constant-effort cycling. SpO_2_ was lower, while heart rate and ratings of perceived difficulty breathing were higher under HY, compared to NM, at all time points (*p* < 0.05). During the constant-effort cycling, heart rate, overall perceived discomfort, difficulty breathing and limb discomfort increased with time (all *p* < 0.05). All variables (except S_p_O_2_) increased along with sense of effort during the brief progressive cycling bouts (all *p* < 0.05). During the two maximal cycling bouts, ratings of overall peripheral discomfort displayed an interaction between time and condition with ratings higher in the second bout under HY *vs*. NM conditions. Conclusion: During self-selected, constant-effort and brief progressive, sub-maximal, and maximal cycling bouts, mechanical work is regulated in parallel to the sense of effort, independently from peripheral sensations of discomfort.

## Introduction

Since its proposal in the early 1920's, the muscle/anaerobic/catastrophic model of exercise capacity has been the focus of research exploring the mechanisms of fatigue during exercise in humans (Fitts, 1994; Bassett and Howley, 2000; Noakes, 2000). These studies have aimed to identify a single factor as the cardinal terminator of exercise, implicating factors such as lactate, phosphate, ammonia, potassium, temperature, and pH (Edwards, 1983; Bassett and Howley, 2000; Noakes and St. Clair Gibson, 2004; Levine, 2007; Amann, 2011; Girard et al., 2011). However, despite the suggestion by Angelo Mosso in 1920 that “nervous fatigue is the preponderating phenomenon, and muscular fatigue also is at bottom an exhaustion of the nervous system” (Bainbridge, 1919) it has taken over a century for the role of the central nervous system (CNS) to again become the focus of exercise performance regulation.

Many integrative models of exercise fatigue now focus on the CNS as being an important regulator of performance; at the core of many of these models is the concept of fatigue as a sensation or emotion (Noakes, 2004a; Lambert, 2005; Tucker, 2009; de Morree and Marcora, 2010; Lane et al., 2011; Swart et al., 2011). In fact, several current models suggest that the rating of perceived exertion (RPE) is of critical importance in dictating central motor drive, and ultimately mechanical output (Noakes, 2004b; Tucker and Noakes, 2009; Marcora and Staiano, 2010). However, despite increasing support for the role of RPE [also referred to by some as the sense of effort or perception of effort (Amann et al., 2007; Dempsey et al., 2008; Marcora, 2009a)] in regulating exercise performance (Edwards, 1983; Bassett and Howley, 2000; Noakes and St. Clair Gibson, 2004; St. Clair Gibson et al., 2006; Levine, 2007; Crewe et al., 2008; Joseph et al., 2008; Tucker, 2009; Amann, 2011; Girard et al., 2011), it is still debated whether this perception or sense is generated via the feedback of afferent sensory receptors stimulated in response to fatiguing locomotor muscles and other organs, and/or is a centrally-originating signal, and whether it acts on the CNS at conscious or sub-conscious levels (Bainbridge, 1919; Marcora, 2009a,b, 2010; Meeusen et al., 2009; Amann and Secher, 2010; Perrey et al., 2010).

Much of the debate over the origin of this sensation of fatigue may be attributed to a too-broad operational definition of the RPE, the interchangeable use of the terms “effort” and “exertion,” inconsistent instructions provided by the researchers to the subjects on how to rate one's own perceived exertion and the selective interpretation of results that incorporate the rating (Noakes, 2004a; Lambert, 2005; Meeusen et al., 2009; Tucker, 2009; de Morree and Marcora, 2010; Lane et al., 2011; Swart et al., 2011; Smirmaul, 2012). In fact, Borg's own evaluation of the rating suggested that it integrates signals not only from peripheral working muscles, the heart and lungs, but also the CNS (Borg, 1982; Noakes, 2004b; Tucker and Noakes, 2009; Marcora and Staiano, 2010). As such, arguments that the rating of perceived exertion is based on a centrally-originating signal only (Amann et al., 2007; Dempsey et al., 2008; Marcora, 2009a) and that it is independent from afferent feedback (Marcora, 2010) are difficult to reconcile. However, these debates highlight the necessity to clarify the distinction between those ratings aimed at evaluating; (1) an internal sense of effort, (2) a perception of peripheral discomfort or (3) an integrated measure of the sum of all signals (both peripherally generated and centrally originating). Despite a recent call to “identify the historical origin of the confusion and to clarify the difference between the sense of effort, other specific sensations and their likely mechanisms” (Smirmaul, 2012), currently little experimental evidence exists to support the claim that the sense of effort (i.e., subjective awareness of effort expended) is distinguishable and independent from perceptions of discomfort arising via afferent feedback.

The aim of the current investigation was therefore to determine if the sense of effort (classified as an evaluation of the subjective awareness of effort expended during a given task) can be distinguished from perceptions of peripheral discomfort, and, more specifically, if muscle activation [assessed via surface electromyography (EMG) signals—a reasonable proxy for net motor unit activity] and subsequent power output produced at a given sense of effort are adjusted in response to altered perceptions of peripheral discomfort. With hypoxia (HY: a reduction in environmental oxygen availability) known to exacerbate the rate of peripheral fatigue development and increase peripheral sensations of difficulty breathing and limb discomfort during both endurance (Amann et al., 2006; Katayama et al., 2006) and sprint exercise (Billaut et al., 2013), it provides a relevant tool to explore the relationship between the perceptions of peripheral discomfort and the sense of effort. As such, we sought to test the proposed disassociation between the sense of effort and perceived peripheral discomfort by “clamping” the sense of effort during self-selected (power output was free to vary), constant-effort cycling and brief progressive sub-maximal and maximal cycling bouts while exploring the influence of HY on adjustments in the subjective perceptions of peripheral discomfort and/or exercise capacity. We hypothesized that HY would increase perceptions of peripheral discomfort leading to a reduction in muscle activation and power output for a given sense of effort.

## Methods

### Participants

Eight, trained, team-sport athletes (minimum of 3 × ~90 min sessions of high-intensity intermittent exercise per week), volunteered for this study (mean ± *SD* age 30.3 ± 2.8 y, stature 1.82 ± 0.58 m, body mass 81.9 ± 7.3 kg). All participants gave written, informed consent before the commencement of the study after all the experimental procedures, associated risks and potential benefits of participation had been explained. The study was approved by the Victoria University Human Research Ethics Committee. All procedures conformed to the Declaration of Helsinki. Participants were asked to avoid vigorous exercise for 24 h, caffeine for 12 h, and food for 2 h before every trial.

### Experimental design

Each participant performed one familiarization session and two experimental trials in a randomized, single-blind design. Participants reported to the laboratory 1 week prior to the first experimental session where they were familiarized with cycling on the SRM cycle ergometer (Schoberer Rad Meßtechnik, Jülich, Germany) and for the determination of their optimal cycling sprint cadence (the pedalling rate that would allow participants to produce the maximal amount of mechanical work output during the maximal 4-s cycling bout Martin and Spirduso, 2001). Briefly, participants completed two sets of 5 maximal 4-s isokinetic cycling sprints on the SRM cycle ergometer at a series of randomized pre-determined sprinting cadences ranging from 100 to 140 rpm with cycling sprint separated by 3 min of passive recovery. The one revolution peak power output for each sprint was recorded via the SRM torque software (Version 12.98 SRM GMBH) and a parabolic curve fitted to the peak power of the 5 sprints, with the optimal cycling cadence being determined as the highest predicted peak power output using the equation of best fit for any given cadence. During this preliminary visit, particular attention was paid to the various modified Borg CR10 scales and the distinction between sensations of effort and perceptions of peripheral discomfort (see *sense of effort and perceived discomfort scales* below).

During the two experimental trials, participants performed two exercise tasks (see *exercise protocol*) under either acute normoxic (NM; simulated altitude/fraction of inspired O_2_ [FiO_2_]: 0 m/0.21) or hypoxic (HY; ~4000 m/0.13) conditions. All tests were completed in a normobaric hypoxic chamber (Colorado Mountain Room System: Colorado Altitude Training, Boulder, CO). Trials were separated by at least 5 days and performed at the same time of day to avoid possible effects of circadian rhythm (Racinais et al., 2010).

### Exercise protocol

Resting heart rate (HR) and arterial oxygen saturation (SpO_2_) were measured prior to, and 10 min following entry to the hypoxic chamber, during which time participants rested in a seated position (wash-in period). Afterwards, they completed 5 min of continuous cycling on the SRM ergometer in the open-end mode at a subjective “sense of effort” of 3 on a modified Borg CR10 scale (see *sense of effort and perceived discomfort scales* below). This was followed after 1 minute by five 4-s submaximal cycling bouts in the isokinetic mode at the individual pre-determined optimal sprinting cadence (group average: 120.7 ± 1.9 rpm). The isokinetic mode allows the subject to pedal without resistance up to the fixed cadence, while resistance is automatically and proportionally increased when participants try to overcome it (Fernández-Peña et al., 2009). In order to carefully examine whether power output produced at a given sense of effort would be adjusted in response to altered perceptions of peripheral discomfort, the same cycling cadence was used for each 4-s cycling bout in all trials. Using this approach, exercise-induced changes in physiological and perceptual responses along with mechanical output would not be influenced by changes in pedalling rates (Elmer et al., 2012). For each of the five submaximal cycling bouts subjects were instructed to work at a subjective “sense of effort” of 4, 5, 6, 7, and 8 on the modified Borg CR10 “sense of effort scale” (see *sense of effort and perceived discomfort scales* below), respectively, with 40 s of recovery (15 s of passive rest and 25 s of cycling at ~100 W) interspersing each bout. After an additional 3 min of recovery (2 min of passive rest and 1 minute of cycling at ~100 W), two 4-s maximal cycling bouts at a subjective “sense of effort” of 10 were completed with each maximal bout being separated by 3 min of recovery (2 min of passive rest and 1 min of cycling at ~100 W). All cycling bouts were initiated from a rolling start, with participants instructed to progressively increase to a cadence within 2–5 rpm of their optimal sprinting cadence during the 10 s prior to each bout. This procedure was used to ensure that all bouts began with the same kinetic energy, while minimizing any jolting sensation as participants reached their optimal sprinting cadence and the breaking resistance was applied. Participants were routinely reminded (~15 s before each cycling bout) with identical instructions to exercise at the prescribed “sense of effort” with the modified Borg CR10 “sense of effort scale” always being visible (see *sense of effort and perceived discomfort scales* below). HR, SpO_2_ and overall perceived discomfort, limb discomfort and difficulty breathing were recorded and reported respectively in an invariant order at 1 minute intervals during the 5 min of constant effort cycling (beginning 45 s into the exercise task) and at exactly 10 s following each 4-s, cycling bout. Subjects were instructed to reflect on their perceptions of discomfort during the preceding exercise bout.

### Physiological responses to exercise

HR and SpO_2_ were monitored and estimated, respectively, via a wireless Polar monitoring system (Polar Electro Oy, Kempele, Finland) and non invasive pulse oximetry using a finger probe (Palmsat 2500, NONINMedical Inc., Plymouth, MI, USA).

### Sense of effort and perceived discomfort scales

Participants were instructed that the “sense of effort” scale is used to set the level of subjective awareness of effort expended during each exercise task. The illustration that “a brief maximal effort requires a maximal conscious effort despite only inducing a small amount of peripheral discomfort” was explained to all participants (Smirmaul, 2012). Sense of effort, as well as rating of overall perceived peripheral discomfort, perceived limb discomfort, and perceived difficulty breathing, were recorded based on modified Borg CR10 scales (See Supplementary Material). During the familiarization session all participants were thoroughly instructed on the distinction between the various sensory and perceptual scales. Specifically, participants were instructed that the “perceived discomfort” scales are used to evaluate their subjective perception of (1) overall peripheral discomfort, (2) specific limb discomfort and (3) difficulty breathing. The questions: “How uncomfortable do you feel overall?,” “How uncomfortable does it feel to breathe?” and “How uncomfortable do your legs feel?” were printed above a modified Borg CR10 scale and visible to participants at all times (See Supplementary Material).

### Electromyography

Electromyographic (EMG) signals from superficial *rectus femoris, vastus lateralis, and vastus medialis* muscles of the right lower limb were recorded using pre-amplified bi-polar surface EMG (Delsys, Trigno Wireless, Boston, Massachusetts, USA) with an interelectrode (center-to-center) distance of 20 mm and placed according to SENIAM's recommendations. Before electrode placement, the skin was lightly abraded and washed to remove surface layers of dead skin, hair, and oil. The ground electrode was attached to the pisiform bone of the right hand. The position of the EMG electrodes was marked with indelible ink (and pictures of the locations were taken) to ensure that they were placed in the same location during subsequent trials. The myoelectric signal was amplified (gain = 1000 x) and filtered (bandwidth frequency = 12–500 Hz) to minimize extraneous noise and possible movement artifacts in the low-frequency region and to eliminate aliasing and other artifacts in the high-frequency region. EMG signals were recorded (sampling frequency = 2000 Hz) using a dedicated analysis system (Spike2 v3.21; Cambridge Electronic, Cambridge Design, Cambridge, UK).

### Data analysis

All power data was analyzed using SRM torque analysis software (SRM Torque Win 1.1.0, SRM, Schoberer Rad MeQtechnik, Jülich, Germany) while all EMG data post-processing was performed in Spike2 (Version 3.21; Cambridge Electronic, Cambridge Design, Cambridge, UK). During the uninterrupted, 5-min cycling exercise the mean power output and corresponding root mean square (RMS) activity for each muscle were recorded at 1-min intervals (average of 8 consecutive cycle revolutions). Regarding sub-maximal and maximal 4 s efforts, the mean power output and RMS activity for the 8 highest cycle revolutions was recorded for each muscle. All power data is expressed as absolute (raw) values (Watts). The average sum of RMS activity of the three muscles was calculated (i.e. quadriceps RMS activity) to provide an index of overall quadriceps muscle activation (Billaut and Smith, 2009; Billaut et al., 2011) and was expressed as a percentage of the maximal RMS activity produced during the 2 highest maximal cycling bouts achieved in any condition (Billaut et al., 2013; Faiss et al., 2013). The neuromuscular efficiency was also calculated as the ratio between the power output and the quadriceps RMS activity.

### Statistical analysis

Statistical analyses were performed using SPSS (version 19.0, Chicago, IL). Separate two-way analyses of variance (ANOVA), with repeated measures for condition (NM vs. HY), time [(minute 1, 2, 3, 4 vs. 5), (effort 4, 5, 6, 7 vs. 8 bouts) or (first vs. second effort 10 bout)] and possible interaction between these two factors was used to test for differences in physiological and perceptual responses for the submaximal continuous, progressive and maximal cycling efforts. Mauchly's test was used to assess for sphericity and in case of violation Greenhouse-Geisser epsilon correction was used to adjust degrees of freedom. When ANOVA revealed a significant main effect, pairwise comparisons were made using the Bonferroni method. Data are presented as means ± SD in the text and means ± s.e.m. within all figures. For each ANOVA, effect size (ES) was calculated (Cohen's d) with the following criteria: an ES of <0.2 is classified as a “trivial,” 0.2–0.4 as a “small,” 0.5–0.7 as a “moderate,” and >0.8 as a “large” effect (Cohen, 2013). Statistical significance was set at *p* < 0.05.

## Results

### Five minutes of constant subjective effort cycling (Figure 1)

During the 5 min of sub-maximal, constant, subjective-effort cycling there was no significant interaction between condition and time for any parameter (all *p* ≥ 0.453). There was no effect of time or condition on the self-selected power output, quadriceps RMS activity, or neuromuscular efficiency (all *p* ≥ 0.271).

**Figure 1:**
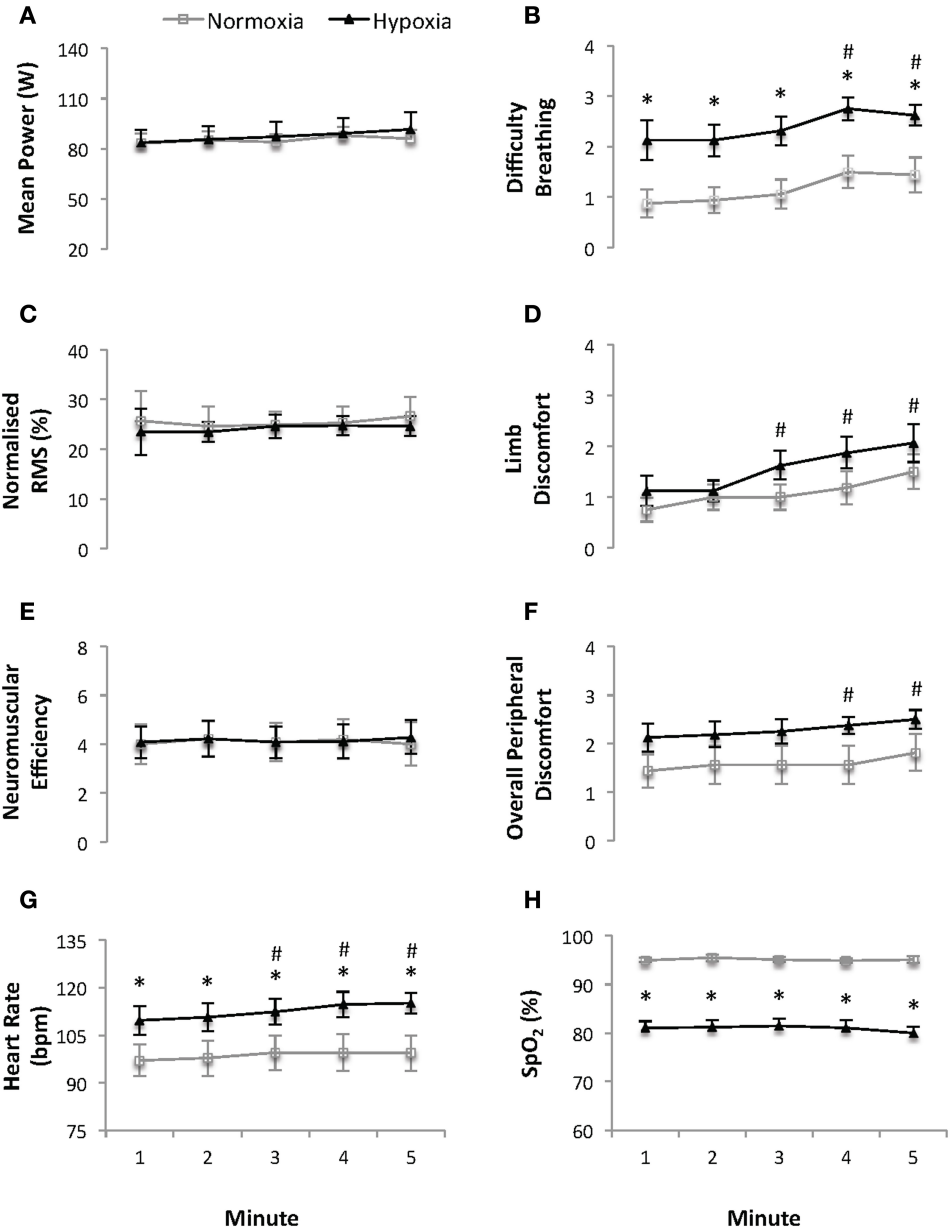
**Mean power (W) (A), difficulty breathing (B), normalized quadriceps Root Mean Square (RMS) electromyographical activity (C), limb discomfort (D), neuromuscular efficiency (au) (E), overall peripheral discomfort (F), heart rate (bpm) (G) and arterial oxygen saturation (SpO_2_) (H) recorded at 1-minute intervals during the 5 min of continuous constant-effort cycling (sense of effort of “3” on a modified Borg CR10 scale; see Supplementary Material)**. Values are mean ± s.e.m., *N* = 8. Data is presented for normoxia (FiO_2_ 0.21) and hypoxia (FiO_2_ 0.13). ^*^ Significant difference vs. normoxia (*p* < 0.05). # Significant difference vs. min 1 (*p* < 0.05).

HR was higher and SpO_2_ lower (both *p* ≤ 0.013, *ES* = 1.03 and 4.43, respectively) at each time point under HY vs. NM conditions. Averaged (5 min) HR and SpO_2_ values were 113 ± 12 bpm and 81.0 ± 4.2% vs. 99 ± 16 bpm and 95.1 ± 1.6% under HY vs. NM conditions, respectively. HR (*p* = 0.019), but not SpO_2_, increased with time independently of the condition.

There was a main effect of time on the rating of overall perceived discomfort, difficulty breathing and limb discomfort (all *p* ≤ 0.001), while only difficulty breathing was higher under HY vs. NM conditions (average for the 5 min of constant subjective effort cycling: 1.2 ± 0.8 and 2.4 ± 0.8 points, respectively; *p* = 0.007, *ES* = 1.49).

### Progressively increasing sub-maximal and maximal 4-s cycling bouts (Figure 2)

During the progressive, 4-s, cycling bouts all parameters (except SpO_2_) increased with time (all *p* ≤ 0.009). There was no effect of condition on power output, quadriceps RMS activity or neuromuscular efficiency during either the progressive, sub-maximal, cycling bouts or the two maximal 4-s cycling bouts (all *p* ≥ 0.407). Mean power output during the two maximal, 4-s s cycling bouts was 1053 ± 45 vs. 1053 ± 57 and 1053 ± 54 vs. 1066 ± 49 W for the first and second bout under HY vs. NM conditions, respectively.

**Figure 2:**
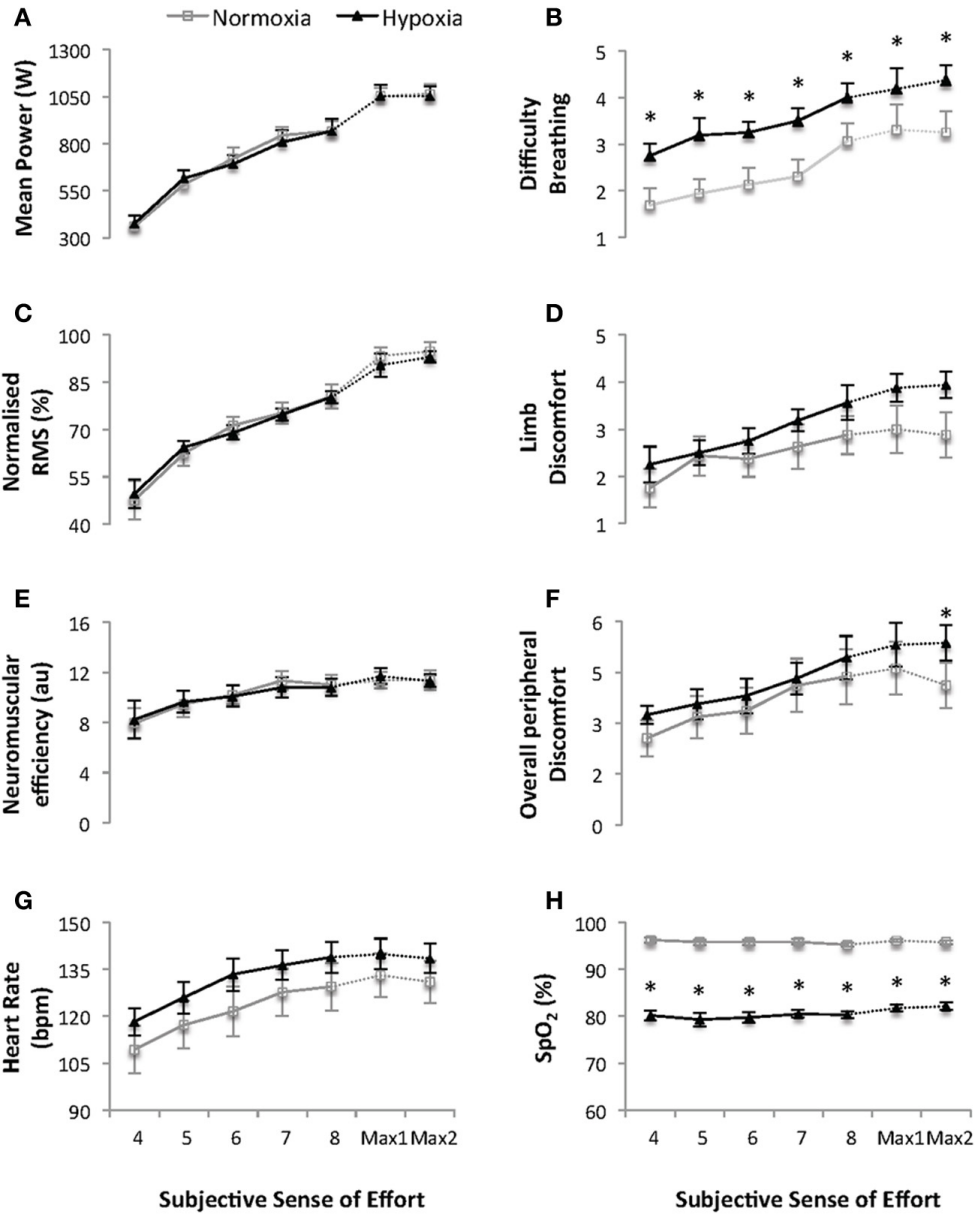
**Mean power output (W) (A), difficulty breathing (B), normalized quadriceps Root Mean Square (RMS) electromyographical activity (C), limb discomfort (D), neuromuscular efficiency (au) (E), overall peripheral discomfort (F), heart rate (bpm) (G) and arterial oxygen saturation (SpO_2_) (H) for the five progressive and two maximal 4-s cycling bouts (sense of effort of “4, 5, 6, 7, 8, and 10” on a modified Borg CR10 scale; see Supplementary Material)**. Values are mean ± s.e.m., *N* = 8. Data is presented for normoxia (FiO_2_ 0.21) and hypoxia (FiO_2_ 0.13). Note that there was an interaction between time and condition (*p* < 0.05) for overall perceived exertion during the 2 maximal 4-s cycling bouts. ^*^ Significant difference vs. normoxia (*p* < 0.05).

During the progressively increasing sub-maximal and maximal 4-s cycling bouts there was a main effect of condition on HR and SpO_2_ (all *p* ≤ 0.033). The mean HR and SpO_2_ for the two maximal 4-s cycling bouts were 131 ± 20 bpm and 95.9 ± 0.9 % and 139 ± 14 bpm and 82.8 ± 2.1 % under NM *vs* HY conditions, respectively (both *p* ≤ 0.033, *ES* = 0.46 and 8.11, respectively).

During the progressive, sub-maximal, cycling bouts ratings of overall perceived discomfort, difficulty breathing and limb discomfort all increased across time (all *p* ≤ 0.009), with higher ratings of difficulty breathing under HY vs. NM conditions. (*p* = 0.011). During the two maximal, 4-s cycling bouts there was an interaction between time and condition on the overall perceived peripheral discomfort (*p* = 0.026) with ratings higher in response to the second bout under HY vs. NM conditions, respectively (5.4 ± 1.5 vs. 4.1 ± 1.9, *p* < 0.05, *ES* = 0.76).

## Discussion

### Summary of main findings

Through the use of a “sense of effort clamp,” we independently explored the effect of HY on altering the level of perceived peripheral discomfort, quadriceps muscle activation and power output during both self-selected, sub-maximal, constant-effort cycling and brief progressive, sub-maximal, and maximal cycling bouts. In contrast to our hypothesis, we observed that for a given “sense of effort” the self-selected muscle activation and power output were not different under HY and NM conditions, despite HY exaggerating both the physiological responses to exercise (i.e., HR and SpO_2_) and ratings of perceived difficulty breathing. These results highlight that perceptions of peripheral discomfort are independent from the sense of effort, and suggest that during sub-maximal, constant-effort cycling, and brief, sub-maximal and maximal cycling bouts, power output is regulated in parallel with the sense of effort but independently from perceptions of peripheral discomfort.

### Sense of effort and perceptions of peripheral discomfort

The first major finding of the current investigation was a dissociation between the “sense of effort” (reported as the subjective awareness of effort expended during each exercise task) and the ratings of perceived peripheral discomfort. Several studies have suggested that perceptions of exercise-induced muscle discomfort and pain are independent from sensations of effort (Meeusen et al., 2009; Perrey et al., 2010; Smirmaul, 2012). However, the inconsistent instructions given to participants regarding RPE, as well as ambiguity over distinctions between leg muscle pain and leg muscle discomfort, have led to questionable evidence that the sense of effort may be generated independently from perceptions of peripheral discomfort. For instance, Hamilton et al. (1996) reported that perceptions of muscle pain were rated as lower than sensations of muscle discomfort during an incremental cycling test. However, no explanation was provided as to how the subjects were instructed on the differences between discomfort and pain. As such it remains possible that muscle pain may have been perceived by subjects as being a description of the same sensation of discomfort yet of higher intensity and therefore lower reported ratings are not surprising. Similarly, Cook et al. (1997) observed an absence of any correlation between their measures of “leg muscle pain” and “leg muscle exertion” during cycling. Their use of RPE was based on the American College of Sports Medicine (ACSM) guidelines for exercise testing. However the “current comment” from the ACSM states that the RPE scale measures feelings of “effort, strain, discomfort, and/or fatigue” and “incorporates information from the internal and external environment of the body” (Utter et al., 2014). Consequently, in the study by Cook et al. (1997), their measures of “pain” and “exertion” may have likely both been influenced by perceptions originating via afferent feedback. As such, it is unclear if their measure of “effort” was independent of peripheral input. In the current investigation we observed increases in overall perceived discomfort, perceived difficulty breathing and perceived limb discomfort during the 5 min of sub-maximal, constant, subjective-effort cycling. Furthermore, higher ratings of perceived difficulty breathing were observed at every time point during the 5 min of sub-maximal, constant, subjective-effort cycling as well as following each brief progressive, sub-maximal and maximal cycling bout, under HY compared to NM conditions. Given the fact that the sense of effort was clamped across conditions, together these findings highlight the disconnect between the sense of effort and perceptions of peripheral discomfort.

In contrast to those attempting to use RPE as a measure of central effort only, Swart et al. (2011) modified the traditional 15-point RPE scale to include the physical sensations experienced by the subject only. To the best of our knowledge, this study provides some of the only, strong evidence that the subjective awareness of effort required to perform an exercise task can be dissociated from the physical discomfort induced by exercise. Through the use of a novel measure of mental effort and their modified RPE scale, which aimed to “include only the physical sensations experienced by the subject”, strong correlations were observed between the task effort awareness, suggested as the psychic effort required to maintain the physical effort, and physical ratings of perceived exertion during a progressive exercise test (*r* = 0.94; Swart et al., 2011), while only moderate correlations were observed during maximal 1-km efforts (*r* = 0.50). These results are in line with our findings of 1) increases in perceptions of peripheral discomfort under both NM and HY conditions during 5 min of sub-maximal cycling while maintaining a constant “sense of effort” and 2) greater perceived difficulty breathing under HY compared to NM conditions during both the 5 min of sub-maximal, constant, subjective-effort cycling and progressive, sub-maximal, and maximal cycling bouts for a given sense of effort. Together, our findings suggest that the subjective awareness of effort expended during a given task (i.e., the sense of effort) may be generated independently from sensations that arise from peripheral afferent inputs (i.e., perceived peripheral discomfort).

The distinction between the sense of effort and perceptions of peripheral discomfort can be well illustrated when considering a brief (< 10 s) maximal effort (Smirmaul, 2012). This task, by nature, requires a maximal “sense of effort” despite perceptions of perceived discomfort being quite low, presumably in accordance with low sensory inputs from peripheral sources such as the heart, lungs and active muscles (Glaister et al., 2005; Billaut and Smith, 2010). However, if this brief maximal effort is repeated, with incomplete recovery, perceptions of peripheral discomfort increase (as reported by RPE), despite a constant required sense of effort (i.e., it is still maximal) (Glaister et al., 2005; Billaut and Smith, 2010). While this example clearly highlights the disconnect between the sense of effort and the perceptions of peripheral discomfort, it also reiterates the origin of confusion surrounding what RPE actually measures (Perrey et al., 2010). In fact, reports of increasing RPE during repeated short duration, maximal-intensity, exercise bouts, in conjunction with decreases in power output and muscle activation (EMG activity), (Glaister et al., 2005; Billaut and Smith, 2010) highlight the tendency of many participants (presumably due to investigator instructions) to score RPE based on perceptions of discomfort only and not incorporating the subjective awareness of effort expended during each bout within their rating. Consequently, some investigators have recognized the limited emphasis that is placed on the conscious sense of effort within current usage of the RPE scale and contradictorily began to argue that RPE is the same as the perception of effort (originally named the sensation of innervation Ross and Bischof, 1981) and completely independent from sensations of discomfort experienced during exercise (Marcora, 2009a; Smirmaul, 2012). However, this antagonistic approach fails to comply with Borg's originally description that RPE represents a “subjective feeling that would represent the sensation originating from the sum of all the bodily systems during exercise” (Borg, 1970). Considering the current debate over what RPE measures, our finding of a distinction between the sense of effort and perceptions of peripheral discomfort emphasizes the need to incorporate separate measures for the “sense of effort” and “perceptions of peripheral discomfort” in place of RPE (which is an integrated measure of the two) in research studies dealing with exercise performance regulation.

### Self-selected power output is regulated in parallel to the sense of effort

During both the 5 min of sub-maximal, constant, subjective-effort cycling and the progressive, sub-maximal, and maximal cycling bouts quadriceps muscle activation and self-selected power output was not different between NM and HY. Additionally, and as previously described during the 5 min of sub-maximal, constant, subjective-effort cycling, this occurred in parallel with increases in physiological (heart rate) and perceptual (ratings of overall perceived discomfort, difficulty breathing and limb discomfort) responses. Ratings of perceived difficulty breathing were also higher in the HY trial at every time point despite muscle activation and power output remaining unchanged across time. Therefore, and in contrast to our hypothesis, another novel finding is that during sub-maximal or maximal, short-duration (4-s) exercise, power output appears to be consciously regulated in parallel to the sense of effort (presumably a centrally-originating signal) produced during the given task and independently from alterations in physiological responses and perceived discomfort that accompanied the task. The sense of effort has been presented as being generated via efferent copies of a centrally-originating signal (i.e., corollary discharge) (Proske, 2005). This suggestion has been based on the premise that “motor areas in the brain would directly influence sensory areas, producing sensations independently of afferent sensory feedback” (Ross and Bischof, 1981).

To date, however, limited evidence of this neurobiological mechanism has been provided to conclusively accept or reject this theory. Arguments in favor of a central sense of effort include: (1) the observations that both peripheral anaesthesia and electrical stimulations result in increased and decreased perception of heaviness, respectively, in accordance with changes in the magnitude of descending motor command during weight lifting tasks (Gandevia and McCloskey, 1977); (2) an increase in the perception of effort required to perform a dynamic cycling task in response to partial neuromuscular block and subsequent higher central command (Gallagher et al., 2001); and (3) higher reported ratings of perceived exertion following a 5-km time trial under conditions of attenuated afferent feedback through the use of subarachnoid injection of fentanyl into the cerebrospinal fluid (Amann et al., 2009). Some of the first direct neurophysiological evidence of this efferent copy of collorary discharge has been provided through the use of electroencephalography during dynamic elbow flexions under fatigued and control conditions (de Morree et al., 2012). It was observed that the “RPE” (interpreted as “how hard they (participants) had to drive their arm to lift the weight”) was correlated with movement-related cortical potentials (measured via electroencephalography). Together, our findings of unchanged muscle activation and power output across time (during the 5 min of sub-maximal, constant, subjective-effort cycling) or condition (during the 5 min of sub-maximal, constant, subjective-effort cycling and the progressive, sub-maximal, and maximal cycling bouts) despite alterations in physiological and perceptual responses supports the suggestion that the “sense of effort” may originate from a signal of central origin and represent the conscious awareness of the central motor command sent to the active muscles (de Morree et al., 2012). However, it is important to note that under conditions of exacerbated peripheral fatigue (e.g., HY), an increased level of muscle activation will be required to produce the same level of force output due to impaired excitation-contraction coupling [reduced neuromuscular efficiency (Hautier et al., 2000)]. Therefore, an increased corollary discharge and associated higher “sense of effort” may occur despite power output being maintained or even reduced. As such, parallel changes in effort and power output may not be valid support that the sense of effort is generated by corollary discharge under conditions of altered neuromuscular efficiency (although this was no the case in the current investigation).

### Preserved maximal exercise capacity

In line with previous research (Smith and Billaut, 2010; Bowtell et al., 2013), we observed well-preserved quadriceps muscle activation and associated power output during the two 4-s maximal cycling bouts under HY and NM conditions, which has been attributed to an enhancement of the anaerobic energy supply during isolated, all-out exercise bouts in acute HY (Ogawa et al., 2005; Morales-Alamo et al., 2013). These results, together with a greater overall perceived peripheral discomfort and perceived difficulty breathing during the two maximal-effort cycling bouts, suggest that perceptions of peripheral discomfort may not be the major contributor to exercise regulation during brief maximal-effort cycling bouts. One explanation may be that despite various ratings of difficulty breathing being higher at each time point or exercise increment, and ratings of overall peripheral discomfort being greater during the second maximal 4-s cycling bout under hypoxic conditions, the actual reported values were quite low. In fact, while anaerobic glycolysis is known to provide the majority of energy during short-duration maximal efforts, it has been shown that the accumulation of metabolic by products believed to evoke sensations of muscle pain and fatigue (e.g., ATP, pH and lactate Pollak et al., 2014) are far from maximal after only one, maximal 6-s cycling sprint (Gaitanos et al., 1993). This may suggest that despite the hypoxic stimulus exacerbating both physiological and perceptual responses, the actual level of hypoxia-mediated, exercise-induced fatigue was insufficient to have a significant impact on the conscious regulation of muscle power output and associated muscle recruitment (EMG). Additionally, a second explanation could be that during brief maximal-effort exercise a higher level of perceived peripheral discomfort can be tolerated or a similar incoming afferent signal is perceived as being less uncomfortable. This suggestion is based on the conscious knowledge/belief that the discomfort will only be brief and will be rapidly removed upon exercise cessation, and may be explained by the psychobiological model of exercise performance (Marcora, 2010). According to this model, an individuals knowledge of the task duration completed and remaining as well as previous experience of perceived exertion during exercise of varying intensity and durations are of critical importance in determining the conscious self-regulation of effort and ultimately power output (Marcora, 2010; Smirmaul et al., 2013). In fact, it has been shown that participants were able to produce the same mean power output during the first 5 s of a 15-s all-out cycling test compared to a 5-s all-out cycling test despite reporting higher RPE during the 15-s all-out test (Wittekind et al., 2011). However, when 30-s and 45-s all-out tests were completed mean power output was lower during the initial 5 seconds compared to the 5-s test; interestingly RPE was not greater during the 30-s test than the 15-s test. Although direct scientific evidence is lacking, we postulate that perceptions of peripheral discomfort (via afferent feedback) may only play a role in the regulation of muscle activation via its influence on the conscious amount of effort given during exercise of certain duration.

Limitations: One potential limitation in the current investigation was the time delay (10 s) between the completion of each 4-s cycling bout and when the participants reported their perceptions of overall peripheral discomfort, difficulty breathing and limb discomfort. This time delay was chosen in order to allow participants time to reflect on their sensations of discomfort, but we acknowledge the fact that since these measurements were taken 10 seconds into the recovery period this may have prevented us from capturing the true magnitude of discomfort experienced during the exercise task. As such, higher ratings may have been reported if participants were required to make an immediate report when exercise stopped. However, despite this we were able to observe differences in individual perceptions of discomfort under hypoxic conditions despite participants exercising at the same “sense of effort” leading to our main contention that despite alterations in physiological and perceptual responses (under hypoxic conditions) for a given sense of effort muscle EMG activity and power output were not different.

## Conclusion

Through the use of a “sense of effort clamp,” our findings suggest that during self-paced, sub-maximal, constant, subjective-effort cycling and brief progressive, sub-maximal, and maximal cycling bouts power output and associated quadriceps muscle activation is regulated in parallel to an internal signal (sense of effort), independently from alterations in physiological responses (presumably higher levels of afferent feedback) and perceived discomfort that accompany the task. Our data therefore suggest that the sense of effort required to complete a given non-exhausting task, and the accompanying perceptions of perceived discomfort, are distinct mechanisms that can both be independently distinguished and individually reported.

### Conflict of interest statement

The authors declare that the research was conducted in the absence of any commercial or financial relationships that could be construed as a potential conflict of interest.
