# Supplementary material for: The role of sense of effort on self-selected cycling power output
Source: Front Physiol. 2014 Mar 31;5:115. doi: 10.3389/fphys.2014.00115 (PMC3978313; doi:10.3389/fphys.2014.00115)
Supplement: Supplementary file 1 [file Presentation1.PDF]

Appendix A

Phrasing scale used for quantification of **sense of effort**

Please rate your subjective awareness of effort expended using the scale shown on the oscilloscope where 0 indicates no discomfort at all and 10 the maximum tolerable level possible.

How much effort did you give or are you giving compared to a maximal effort?

How hard are you trying

**0**	**No effort**
**0.5**	**Very, very slight (almost none)**
**1**	**Very slight**
**2**	**Slight**
**3**	**Moderate**
**4**	**Somewhat hard**
**5**	**hard**
**6**	
**7**	**Very hard**
**8**	
**9**	**Very, very hard (almost maximal)**
**10**	**Maximal**

Appendix B

Phrasing scale used for quantification of **overall perceived peripheral discomfort**

Please rate your overall perceived peripheral discomfort using the scale shown on the oscilloscope where 0 indicates no discomfort at all and 10 the maximum tolerable level possible.

What is your overall perceived peripheral discomfort

How uncomfortable do you feel overall?

**0**	**Nothing at all**
**0.5**	**Very, very slight (almost none)**
**1**	**Very slight**
**2**	**Slight**
**3**	**Moderate**
**4**	**Somewhat severe**
**5**	**Severe**
**6**	
**7**	**Very severe**
**8**	
**9**	**Very, very severe (almost maximal)**
**10**	**Maximal**

Appendix C

Phrasing scale used for quantification of **perceived difficulty breathing**

Please rate your overall perceived peripheral discomfort using the scale shown on the oscilloscope where 0 indicates no discomfort at all and 10 the maximum tolerable level possible.

What is your perceived difficulty breathing

How uncomfortable does it feel to breathe?

**0**	**Nothing at all**
**0.5**	**Very, very slight (almost none)**
**1**	**Very slight**
**2**	**Slight**
**3**	**Moderate**
**4**	**Somewhat severe**
**5**	**Severe**
**6**	
**7**	**Very severe**
**8**	
**9**	**Very, very severe (almost maximal)**
**10**	**Maximal**

Appendix D

Phrasing scale used for quantification of **perceived limb discomfort**

Please rate your overall perceived peripheral discomfort using the scale shown on the oscilloscope where 0 indicates no discomfort at all and 10 the maximum tolerable level possible.

What is your perceived limb discomfort

How uncomfortable do your legs feel?

**0**	**Nothing at all**
**0.5**	**Very, very slight (almost none)**
**1**	**Very slight**
**2**	**Slight**
**3**	**Moderate**
**4**	**Somewhat severe**
**5**	**Severe**
**6**	
**7**	**Very severe**
**8**	
**9**	**Very, very severe (almost maximal)**
**10**	**Maximal**
